# One Mixture to Rule Them All: Enhancing Efficiency and Standardization of Industrial High-Temperature Heat Pumps

**DOI:** 10.1021/acsengineeringau.4c00060

**Published:** 2025-06-13

**Authors:** Philip Widmaier, Leon P. M. Brendel, Stefan S. Bertsch, André Bardow, Dennis Roskosch

**Affiliations:** † Energy and Process Systems Engineering (EPSE), 27219 ETH Zurich, Tannenstrasse 3, 8092 Zurich, Switzerland; ‡ Institute for Energy Systems (IES), 30441 Eastern Switzerland University of Applied Sciences, Werdenbergstrasse 4, 9471 Buchs, Switzerland

**Keywords:** high-temperature heat pumps, zeotropic mixtures, temperature glide, process heat, standardization

## Abstract

High-temperature heat pumps are preferred for decarbonizing many industrial processes, but are still being adopted slowly. Major barriers to adoption are low efficiency, leading to high operational cost, and the need for custom-made designs, increasing investment cost. In this work, refrigerant mixtures are exploited to overcome these barriers for high-temperature heat pump adoption. Mixtures have been known to improve heat pump efficiency if their nonisothermal phase change is matched to heat source and sink temperature changes. Beyond that, we improve standardization by using mixture composition as an additional degree of freedom to tailor a standard heat pump designed for a specific refrigerant pair to various applications. By model-band screening of 703 refrigerant pairs across 81 combinations of heat source and sink temperature changes, we identify a maximum COP advantage of 26% for a refrigerant mixture when the maximum heat source and sink temperature changes of 40 K occur. Several mixtures are identified yielding near-optimal efficiencies across all 81 heat source and sink temperature changes. The best all-rounder mixture, diethyl ether/cyclopropane, retains, on average, 97% efficiency of the individually optimal mixtures. These findings support the development of more efficient and less costly high-temperature heat pumps, a crucial step in the heat transition.

## Graphical abstract

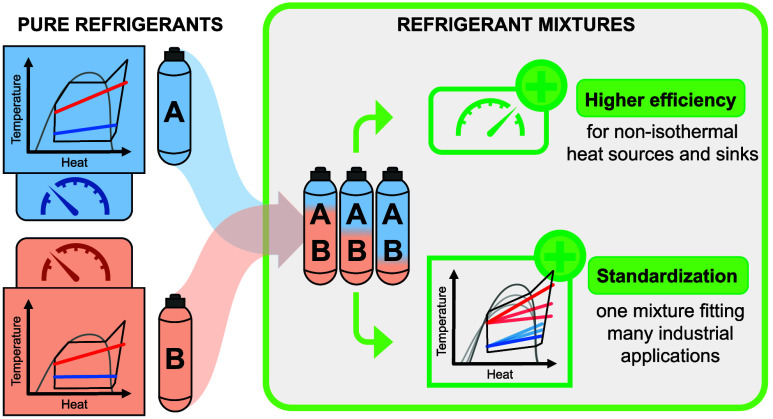

## 1 Introduction

Heat pumps are central in decarbonizing residential and industrial heat supply. In the residential heating sector, heat pumps have become prevalent with a 40% growth in installations in Europe during 2022.^1^ Likewise, governments and international organizations have recognized the high decarbonization potential of high-temperature heat pumps (HTHPs) for industrial process heat: Numerous reports acknowledge HTHPs as a promising source for process heat, and advocate strategies for their implementation.^2–4^ HTHP are regarded as particularly promising for industrial sectors with heat demands within the temperature band of *T* < 200 °C, such as pulp/paper, chemicals, food/beverages, nonferrous metals, and refinery.^5–8^ Guidelines by policymakers, together with the significant market potential of HTHPs, foster intensive research and development in HTHP technology.^5,9^

However, HTHPs currently only supply a minor share of industrial process heat. Industrial processes impose challenging requirements on HTHPs, such as high supply temperatures and large temperature changes in heat source and sink.^10,11^ Current HTHPs only yield moderate efficiencies for these challenging process conditions. Moreover, industrial processes are diverse, and so are their requirements for process heat.^6^ The high diversity in industrial heat demand is currently accounted for by customizing HTHP systems.^12^ The small-scale manufacturing of HTHP systems and a lack of standardization increase investment costs.^9^

A promising approach to increasing both HTHP efficiency and standardization is the use of refrigerant mixtures. Refrigerant mixtures offer additional degrees of freedom: Selecting the pure components determines a range of thermo-physical properties that can be specifically optimized by adjusting the mixture composition. Zeotropic mixtures, in particular, have a unique feature as they change temperature during evaporation and condensation, unlike pure refrigerants. This temperature glide can be used to align the refrigerant’s temperature changes with the heat source and sink (glide matching), thereby reducing exergy losses in the heat exchangers and increasing heat pump efficiency.^13^

The exploitation of the temperature glide to increase heat pump efficiency has been investigated in several studies. Primarily focusing on residential heat pumps, these studies confirm the theory of glide matching and demonstrate the efficiency benefits of zeotropic mixtures with temperature glide.^14–16^ However, the actual efficiency advantages of mixtures over pure refrigerants only range within a few percent.^17,18^ The small efficiency gain from zeotropic mixtures in residential heat pump applications may be attributed to the typically minor temperature changes of residential sources and sinks (5–10 K), reducing the relevance of glide matching.

The situation is different for industrial HTHPs: Industrial heat sources and sinks often undergo significant temperature changes during heat transfer (typically 5–60 K^19^). Thus, glide matching seems particularly attractive for improving heat pump efficiencies.^6^ The COP improvements of high-glide refrigerant mixtures were analyzed for HTHPs in several studies. Zühlsdorf et al.^20^ modeled a standard heat pump cycle and studied four combinations of heat source and sink temperature changes considering a refrigerant mixture set based on 14 natural refrigerants. No advantage of mixtures could be found for small temperature changes of the source (Δ*T*_so_ = *T*_so,in_ – *T*_so,out_ = 5 K). For large heat source and sink temperature changes (Δ*T*_so_ = 20 K, Δ*T*_si_ = *T*_si,out_ – *T*_si,in_ = 40 K), the study identified a COP advantage of 27% for the best refrigerant mixture (dimethyl ether (40 mass-%)/isopentane (60 mass-%)) compared to the best pure refrigerant (isopentane). Additionally, the temperature change of the heat source was shown to impact the refrigerant mixture COP advantage more than the temperature change of the heat sink. Abedini et al.^21^ considered three case studies for refrigerant mixtures in HTHPs, represented by combinations of heat sources and sinks (isothermal and nonisothermal). A considerable impact of glide matching on the COP of mixtures was only observed for a completely sensible heat source (Δ*T*_so_ ≈ 10 K) and sink (Δ*T*_si_ = 60 K), where ethanol/*p*-xylene outperformed the best pure refrigerant methanol by over 10%. For isothermal/latent heat sources, mixtures did not provide significant COP advantages, regardless of the heat sink temperature change. The study of Brendel et al.^22^ experimentally investigated mixtures of R1336mzz­(Z), R1233zd­(E), R1224yd­(Z), R1234yf, and *R*32. Mixtures at various compositions were compared against the pure refrigerants for a heat source inlet temperature of *T*_so,in_ = 60 °C, a heat sink outlet temperature of *T*_si,out_ = 100 °C, and source/sink temperature changes. For heat source and sink temperature changes of Δ*T*_so_ = Δ*T*_si_ = 35 K, a refrigerant mixture (R1336mzz­(Z)/R1234yf) increased COP up to 16% compared to the best-performing pure refrigerant (R1234yf). Based on further measurements, Brendel et al. conclude that good glide matching at heat source and sink temperature changes of 35 K could enable COP improvements of up to 37% assuming sufficiently large heat exchangers.^23^ Analogous tests on synthetic and natural refrigerants showed propane (70 mass-%)/pentane (30 mass-%) achieving the largest COP increase of 19% relative to the best pure refrigerant (butane).^24^

Present studies on high-glide refrigerant mixtures suggest a considerable efficiency potential for industrial HTHPs. However, the models and experiments are restricted to a small number of case studies and a sparse refrigerant set. Furthermore, model studies often neglect the influence of refrigerant-dependent compressor efficiency that was shown to be particularly decisive for the evaluation of mixture COPs.^25,26^ The potential for improving HTHP efficiency through the use of refrigerant mixtures with temperature glide has not yet been systematically explored across the wide variety of industrial process conditions.

While the potential of refrigerant mixtures to improve efficiency has been recognized, mixtures could be a breakthrough technology for standardizing industrial heat pumps: Adjusting a mixture’s thermo-physical properties and the temperature glide through composition allows for convenient tailoring to various applications without changing the pure components. Given a targeted selection of the pure mixture components, manufacturers can develop a corresponding standardized heat pump design, achieving near-optimal COPs for many applications through composition tailoring. Such a standardized design can reduce investment costs. Standardizing HTHPs through refrigerant pairs that achieve near-optimal efficiency for various process conditions has not been considered to date.

In this contribution, we close the gap by systematically analyzing the potential of zeotropic refrigerant mixtures to increase efficiency and standardize industrial HTHPs. We screen an extensive refrigerant set for various heat sources and sinks. The screening employs an HTHP process model that optimizes the COP for each refrigerant (pure + mixture). A loss-based compressor model accounts for refrigerant-dependent compressor efficiencies.^27^ In the analysis of the screening data set, we focus on the temperature glide as a key driver for the cycle efficiency of mixtures. From the screening data set, we identify the specialists among refrigerant mixtures that maximize efficiency advantages compared to pure refrigerants, specific to heat source and sink. Furthermore, we target all-rounder refrigerant pairs that yield near-optimal efficiencies across various heat sources and sinks if their composition is adapted.

Our findings provide insights into the process conditions that allow for the efficient use of mixtures and those that do not. High-efficiency all-rounder refrigerant pairs are introduced.

## 2 Methodology

We evaluate the benefits of refrigerant mixtures through a large-scale screening of systematically selected heat source and sink pairings (representing HTHP applications) and a refrigerant set covering a wide range of molecular properties. By optimizing the heat pump process toward the COP for each pure refrigerant/refrigerant mixture and heat source and sink pairing, a comprehensive data set is obtained to evaluate the potential of refrigerant mixtures.

Section 2.1 presents the considered heat pump process, Section 2.2 lists the component models, and Section 2.3 features process optimization, while Section 2.4 introduces the case study.

### 2.1 Heat Pump Process

The study considers a subcritical heat pump cycle with an internal heat exchanger (IHX) as an additional component to the evaporator, compressor, condenser, and throttle (Figure 1). After full condensation and subcooling in the condenser, the liquid refrigerant enters the IHX, and heat is transferred to the fully evaporated refrigerant at the evaporator outlet to provide superheating. The IHX increases the COP for most refrigerants, is comparatively inexpensive to purchase, and is often used in industrial applications.^21,28^ Superheating at the compressor inlet is assumed to be fully provided by the IHX. The calculation scheme of the process’s thermodynamic states (1, 2, 2_s_, 3_sc_, 3, 4) is provided in Table 1.

**Figure 1:**
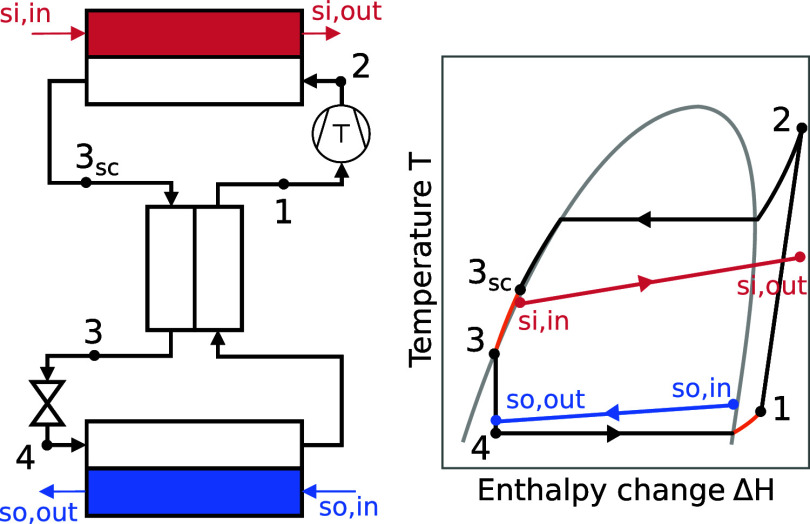
Heat pump flow sheet (left) and temperature-enthalpy diagram (right). States 1, 2, 3sc, 3, 4 refer to the heat pump’s refrigerant, whereas states so,in, so,out and si,in, si,out refer to the heat source and sink at the heat exchanger inlets and outlets, respectively.

**Table 1:** Calculation of the Process Model’s Thermodynamic States Based on the Evaporation Temperature *T*_ev_ and Condensation Temperature *T*_co_ (Defined at the Dew Line, Respectively), Superheating Δ*T*_sh_, and Subcooling Δ*T*_sc_

**thermodynamic state**	**state parameters**	
1	*p*_1_ = *p*_ev_ = *p*_dew_(*T*_ev_)	*T*_1_ = *T*_ev_ + Δ*T*_sh_
2_s_	*p*_2s_ = *p*_co_ = *p*_dew_(*T*_co_)	*s*_2s_ = *s*_1_
2	*p*_2_ = *p*_co_	$h_{2}=\frac{h_{2s}-h_{1}}{\eta_{\mathrm{is}}}+h_{1}$
3_sc_	*p*_3sc_ = *p*_co_	*T*_3sc_ = *T*_bubble_(*p*_co_)– Δ*T*_sc_
3	*p*_3_ = *p*_co_	*h*_3_ = *h*_3sc_ – *h*_1_ + *h*_dew_(*T*_ev_)
4	*p*_4_ = *p*_ev_	*h*_4_ = *h*_3_

We use the heat pump’s COP as the main evaluation metric. The COP is defined as the ratio of the useful heat provided by the heat pump *Q̇*_h_ and the compressor power *P*_comp_.

1 $$\mathrm{COP}=\frac{{\mathrm{Q̇}}_{h}}{P_{\mathrm{comp}}}=\frac{q_{h}}{w_{\mathrm{comp}}}$$

The reversible cycle delineates the thermodynamic cycle efficiency limit (COP^rev^). The reversible cycle’s COP^rev^ can be derived from the heat source’s and sink’s thermodynamic mean temperatures *T* _so_ and *T* _si_, respectively (eq 2, so-called Lorenz COP).

2 $${\mathrm{COP}}^{\mathrm{rev}}={\left(1-\frac{{\mathrm{T̅}}_{\mathrm{so}}}{{\mathrm{T̅}}_{\mathrm{si}}}\right)}^{-1}$$

The heat source’s and sink’s thermodynamic mean temperatures are calculated as the ratio of the mass-specific enthalpy and entropy differences between the respective heat exchanger’s inlet and outlet states assuming isobaric conditions (eq 3).

3 $$\mathrm{T̅}=\frac{h_{\mathrm{out}}-h_{\mathrm{in}}}{s_{\mathrm{out}}-s_{\mathrm{in}}}$$

### 2.2 Component Models

The heat exchangers are modeled as counter-flow and assumed to be isobaric and adiabatic. Following a standard approach in heat exchanger modeling,^29^ we use pinch models to connect process temperatures, such as evaporation and condensation temperatures, and heat source and sink temperatures. Pinch models map the temperature difference between the refrigerant and the secondary fluid (called approach temperature Δ*T*_app_) during heat transfer. Small approach temperatures are favored regarding cycle efficiency but lead to larger heat transfer areas. Restricting a minimum allowed approach temperature ensures efficient heat transfer and reasonably sized heat exchangers. We track the approach temperature at several points along the heat transfer isobar in each heat exchanger to account for the potentially nonlinear temperature glide.^30^

The compressor is modeled using an isentropic compressor efficiency.^29^ The isentropic compressor efficiencies are determined by a loss-based compressor model that, in particular, accounts for refrigerant dependency.^27^ The compressor model’s parameters are refitted using experimental HTHP data to achieve plausible efficiency predictions for industrial applications (consult Supporting Information, Table SI1, for pure refrigerant isentropic compressor efficiencies at reference process conditions). The throttling of the refrigerant after the condenser is assumed to be isenthalpic.

### 2.3 Process Optimization

To adapt the cycle, we optimize the heat pump process for every heat source and sink pairing (gray dots, Figure 2) and refrigerant (mixture) considered. The optimization variables are the evaporation and condensation temperatures *T*_ev_ and *T*_co_ (defined at the dew line, respectively), the superheating at the compressor inlet Δ*T*_sh_, and the subcooling at the IHX inlet Δ*T*_sc_ which are optimized for COP (eq 4).

4 $$\begin{array}{ll} \max_{X} & \mathrm{COP}=f\left(X\right)\\ \mathrm{with} & X=\left[T_{\mathrm{ev}},T_{\mathrm{co}},\Delta T_{\mathrm{sh}},\Delta T_{\mathrm{sc}}\right]\\ s.t. & h\left(X\right)=0\\ & g\left(X\right)\geq 0\\ & X_{\mathrm{lb}}\leq X\leq X_{\mathrm{ub}} \end{array}$$

**Figure 2:**
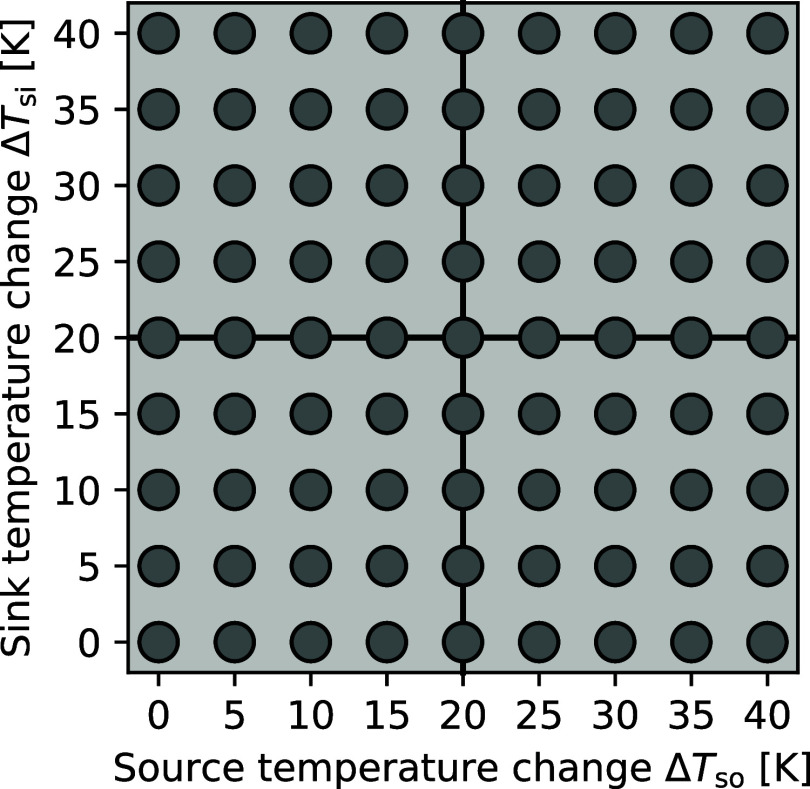
Heat source and sink pairings considered in the study. For *T*_so,in_ = 60 °C and *T*_si, out_ = 100 °C, heat source and sink pairings are configured by varying the heat source and sink temperature changes Δ*T*_so_, Δ*T*_si_ between 0 and 40 K.

The optimization is subject to equality and inequality constraints (*h*(*X*) and *g*(*X*), respectively) and variable bounds *X*_lb_, *X*_ub_ to ensure a feasible heat pump process (eq 4). Inequality constraints are employed, e.g., to keep the minimum approach temperature in the heat exchanger, guarantee dry compression, and limit process pressures. All constraints are summarized in Table 2.

**Table 2:** Process Constraints Employed in the Thermodynamic Cycle Optimization (eq 4)­^a^ 0 K and 40 K

**constraint subject**	**constraint**
condensation temperature	*T*_co_ ≤ *T*_crit_ – 10 K
evaporator pressure	*p*_ev_ ≥ *p*_ev,min_ = 20 kPa
enthalpy during compression	$\begin{array}{l} h_{1}+\frac{h\left(p=p^{*},s=s_{1}\right)-h_{1}}{\eta_{\mathrm{is}}}>h_{\mathrm{dew}}\left(p^{*}\right)\\ \forall p^{*}\in \left[p_{\mathrm{ev}},p_{\mathrm{co}}\right] \end{array}$
approach temperature diff.	$\begin{array}{l} \left|T^{R}-T^{\mathrm{SF}}\right|\geq \Delta T_{\mathrm{app},\min}=5\;K\\ \mathrm{in}\;\mathrm{all}\;\mathrm{heat}\;\mathrm{exchangers} \end{array}$
temperature lift	*T*_co_ > *T*_ev_
compressor outlet temperature	*T*_2_ < *T*_2,max_ = 200 °C

a R: refrigerant, SF: secondary fluid.

The minimum evaporator pressure is often limited to ambient pressure to rule out air suction into the system and ensure high densities at the compressor inlet (associated with high compressor efficiencies). However, constraining the minimum evaporator pressure to 1 bar substantially reduces the number of feasible refrigerants for HTHPs. In the interest of fundamentally exploring the refrigerant mixture potential, we set the minimum allowable evaporator pressure to *p*_ev,min_ = 20 kPa. We believe that evaporation pressures of 20 kPa are manageable in an industrial context and worth pursuing.

To ensure sound process optimization, the optimization variables *X* are limited within thermodynamically reasonable parameter ranges, defined by lower and upper bounds *X*_lb_ and *X*_ub_, respectively (Table 3). The evaporation temperature’s upper bound and the condensation temperature’s lower bound are given by the heat source’s highest temperature *T*_so,in_ = 60 °C and the heat sink’s lowest temperature *T*_si,in_ = 100 °C – Δ*T*_si_, respectively. The condensation temperature’s upper bound ensures a subcritical process, considering a temperature difference of 10 K between the refrigerant’s critical temperature *T*_crit_ and the condensation temperature *T*_co_. The evaporation temperature’s lower bound is set to comply with the process constraint on the evaporator pressure *p*_ev_. The superheating Δ*T*_sh_ and subcooling Δ*T*_sc_ are both limited by a lower bound of 3 K to ensure full evaporation and condensation; A temperature margin of 25 K establishes the respective upper bounds.

**Table 3:** Optimization Bounds for the Process Optimization Variables

	*T*_ev_	*T*_co_	Δ*T*_sh_	Δ*T*_sc_
upper bound *X*_ub_	*T*_so,in_	*T*_crit_ – 10 K	28 K	28 K
lower bound *X*_lb_	*T*(*p* = *p*_ev,min_, *x* = 1)	*T*_si, in_	3 K	3 K

The process optimization algorithm is written in Python, leveraging the SciPy library’s *minimize* function with the Sequential Least Squares Quadratic Programming (SLSQP) method to address the Nonlinear Programming (NLP) problem.^31^

### 2.4 Case Study

To systematically explore the potential of refrigerant mixtures for various applications, the heat pump process model is evaluated across various heat source and sink pairings. The temperature levels and temperature changes of the heat sources and sinks examined in this work are aligned with process conditions typically encountered in industrial sectors suitable for HTHP integration.^6,10^ Thus, the source inlet temperature and the sink outlet temperature are set to *T*_so,in_ = 60 °C and *T*_si,out_ = 100 °C, respectively. The source outlet temperature *T*_so,out_ and the sink inlet temperature *T*_si,in_ are systematically varied over a range of 40 K in 5 K intervals, resulting in 81 unique heat source and sink pairings *ij* with indices *i* (source), *j* (sink) ∈ [0, 5, 10,..., 40]. The considered heat source and sink temperature changes are typical for heat supply intensive processes in important industries.^19^

We consider a refrigerant set comprising 38 preselected pure substances, from which 703 binary refrigerant pairs are derived (full refrigerant set in Supporting Information, Table SI2). The pure refrigerants cover various substance groups (Table 4) and include both typical refrigerants and substances not yet commercially utilized. We only choose pure refrigerants with an Ozone Depletion Potential (ODP) = 0 and a Global Warming Potential (GWP) < 150. In addition to the pure refrigerants, nine compositions are considered for each binary refrigerant pair from *x*_mol_ = 0.1 ... 0.9 with d*x*_mol_ = 0.1 mol/mol. The overall refrigerant set hence comprises 6365 refrigerants in total. The resulting set of pure refrigerants and mixtures represents a wide range of thermo-physical properties and mixture behaviors, providing a comprehensive basis for mixture assessment. The mixtures yield temperature glides spanning from 0 K to 60 K (Figure 3) and, thus, cover the temperature changes of heat source and sink between 0 K and 40 K.

**Figure 3:**
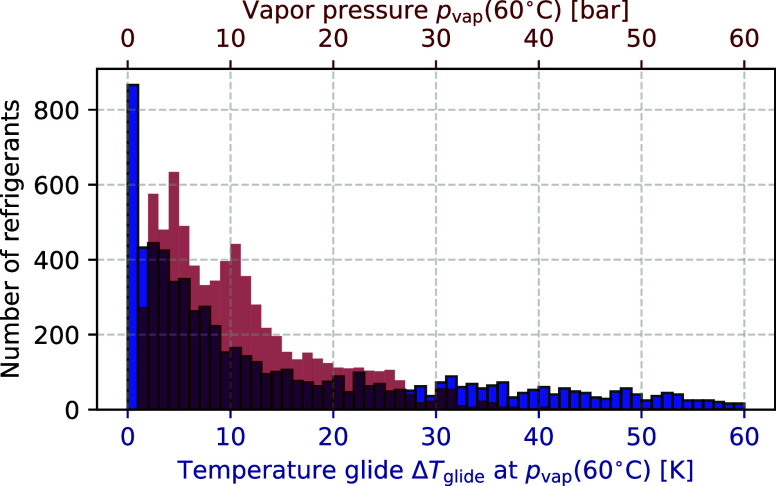
Distribution of temperature glides (0−60 K) and vapor pressures (0−60 bar) for all refrigerants considered in the screening study (pure refrigerants and refrigerant pairs with discretized composition d*x* = 0.1 mol/mol). Vapor pressure *p*_vap_ at a dew line temperature of *T*_dew_ = 60 °C. Temperature glide at vapor pressure *p*_vap_, Δ*T*_glide_ = *T*_dew_ – *T*_bubble_.

**Table 4:** Pure Refrigerant Set by Molecular Family

family	nr. of pure refrigerants
alcohols	2
branched alkanes/alkenes	6
cycloalkanes/-alkenes	4
ethers	2
hydrofluorocarbons (HFCs)	1
hydrofluoroolefins (HFOs)	7
linear alkanes/alkenes	12
others	2
other hydrocarbons	2

All fluid property data is obtained using the REFPROP fluid property database.^32^ Even though REFPROP provides highly accurate pure fluid property data,^24,33^ the authors are aware that high-quality mixing rules might not be available for all refrigerant pairs contained in the refrigerant screening set. This lack of genuine mixing rules might sometimes lead to inaccurate property predictions. Potential inaccuracies in the refrigerant property data underlying the screening study may limit the reliability of detailed statements about individual pure refrigerants and refrigerant mixtures. However, this study primarily aims to explore trends in the efficiency of refrigerant mixtures across a range of typical industrial applications. The primary focus of our study is to investigate mixture performance and derive general guidelines for the efficient use of refrigerant mixtures, with less focus on pinpointing individual mixtures. Thus, we assume that the vast molecular space allows the generation of refrigerant mixtures exhibiting the discussed properties in this work, justifying the drawn conclusions.

### 2.5 Identification of All-Rounder Refrigerants

In principle, all refrigerants, including pure refrigerants, can be applied over various heat source and sink pairings. However, the efficiency can substantially decline since the thermo-physical properties may no longer match the requirements of the process. Binary refrigerant pairs offer a much greater all-rounder potential, as thermo-physical properties (namely the temperature glide) can be tailored between the pure refrigerant’s properties by adjusting the composition. To advance standardization in industrial heat pumps, a standard heat pump design can be adapted to a suitable all-rounder pair (material compatibility, licensing), and subsequently tailored to various industrial heat sources/sinks simply by adjusting the all-rounder’s composition.

We proceed as follows to identify all-rounder refrigerants from the data gathered during the refrigerant screening. The pure refrigerants and refrigerant mixtures that yield feasible heat pump cycles across all heat source and sink pairings are considered all-rounder refrigerants. To evaluate and rank an (all-rounder) refrigerant’s performance for a given heat source and sink pairing *ij*, we introduce the Relative Coefficient of Performance RCOP_*ij*_. The refrigerant’s maximum COP_*ij*_^max^ for a specific heat source and sink pairing *ij* is related to the COP_*ij*_^opt^ obtained for the COP-optimal specialist refrigerant. The COP-optimal refrigerants are selected from all pure refrigerants and mixtures included in the screening.

5 $${\mathrm{RCOP}}_{\mathrm{ij}}=\frac{{\mathrm{COP}}_{\mathrm{ij}}^{\max}}{{\mathrm{COP}}_{\mathrm{ij}}^{\mathrm{opt}}}$$

As a measure of all-rounder performance, we finally consider the Relative COP (RCOP*_ij_*) across all *n* = 81 heat source and sink pairings and determine the refrigerant’s Average Relative Coefficient of Performance $\overset{®}{\mathrm{RCOP}}$ :

6 $$\overset{®}{\mathrm{RCOP}}=\frac{1}{n}\cdot \sum {\mathrm{RCOP}}_{\mathrm{ij}}$$

## 3 Results and Discussion

This section analyzes the refrigerant screening results across all heat source and sink pairings. The analysis focuses on the temperature glide of refrigerant mixtures and its role as a key driver for efficiency improvement. The study’s data set captures various refrigerant properties that influence cycle efficiency and reflect trade-offs, such as evaporation enthalpy and isentropic compressor efficiency. However, the temperature glide is the unique property of mixtures and the key differentiator offering potential for efficiency improvement. Accordingly, we focus on the impact of the temperature glide and discuss the influences of other refrigerant properties only marginally.

First, general trends of the COP as a function of composition are discussed to guide the search for high-efficiency refrigerant mixtures (Section 3.1). Subsequently, specialist mixtures are investigated that deliver the maximum COP for given heat source and sink temperature changes (Section 3.2). Finally, all-rounder refrigerant pairs are targeted that yield near-optimal COPs over a range of heat source and sink temperature changes if the composition is tailored (Section 3.3).

### 3.1 General Trends in Refrigerant Mixture COPs

The heat pump COP highly depends on the mixture composition *x*_mol_. For the heat source and sink pairing of Δ*T*_so_ = Δ*T*_si_ = 25 K (Figure 4), many mixtures yield higher COPs than the COP-optimal pure refrigerant (cyclopropane, COP_25,25_^opt,pure^ = 3.9). The COP-optimal refrigerant pair (diethyl ether/cyclopropane) surpasses the highest pure refrigerant COP by up to 16%.

**Figure 4:**
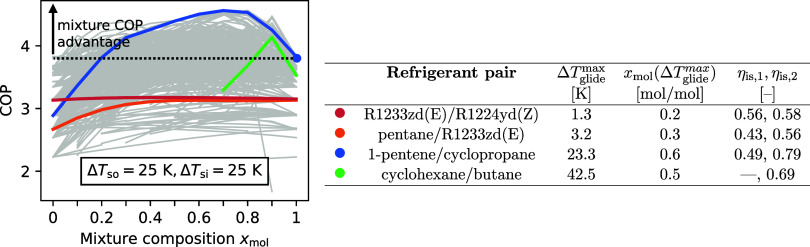
COP as a function of the second mixture component’s mole fraction *x*_mol_ for all refrigerant pairs yielding feasible heat pump processes. Heat source and sink temperature changes of Δ*T*_so_ = 25 K, Δ*T*_si_ = 25 K. Four characteristic COP profiles are highlighted: R1233zd­(E)/R1224yd­(Z), pentane/R1233zd­(E), 1-pentene/cyclopropane, cyclohexane/butane. Cyclopropane (blue dot) yields the highest pure refrigerant COP and sets the reference for the COP advantage of mixtures. The table displays relevant refrigerant pair properties. Maximum refrigerant pair temperature glide at a dew line temperature of 60 °C: Δ*T*_glide_^max^ = max­(*T*_dew_(p*, *x*_mol_) – *T*_bubble_(p*, *x*_mol_)), for p* = *p*_vap_(60 °C) and *x*_mol_ ∈ [0.1,..., 0.9].

We observe four groups of refrigerant pairs sharing the same trends of the COP over composition (Figure 4):

(1) The first group of refrigerant pairs shows no COP improvements over composition while the pure components’ COPs are similar (red line, Figure 4). These refrigerant pairs typically have low temperature glides and the pure components yield similar compressor efficiencies (see Table in Figure 4). R1233zd­(E)/R1224yd­(Z) is a typical example for this group of refrigerant mixtures.
(2) The second group of refrigerant pairs does not significantly improve COP either. However, the pure components’ COPs differ (orange line, Figure 4). These refrigerant pairs exhibit low temperature glides and, therefore, do not provide a significant benefit in COP. The COP differs over composition mainly due to the changing isentropic compressor efficiency between the pure refrigerants (see Table in Figure 4). In close approximation, the compressor efficiency changes linearly with the composition between the pure substance efficiencies.^25^ A typical example of this group of mixtures is pentane/R1233zd­(E).
(3) The third group of refrigerant pairs yields the desired COP increase in the mixture (COP increase of 16% over pure cyclopropane, blue line, Figure 4). The refrigerant pairs are characterized by substantial temperature glides, while the maximum temperature glide closely matches the heat source and sink temperature changes. The pure components’ COPs are high but may differ (see Table in Figure 4). A typical representative is 1-pentene/cyclopropane. According to Roskosch et al.,^25^ even greater COP benefits can be expected if the compressor efficiency remains constant over the mixture, along with a well-matching temperature glide.
(4) The last group of refrigerant pairs is characterized by a maximum temperature glide significantly larger than the heat source and sink temperature changes. At a certain composition, a too-large temperature glide shifts the evaporator pinch point, causing a drop in COP as showcased by cyclohexane/butane (green line, Figure 4). For compositions with even larger temperature glides cycles may not be feasible, truncating the COP profile.

Each refrigerant pair group may include pairs where only one pure component allows a feasible heat pump cycle, while the other violates process constraints (e.g., too low critical temperature). As a result, the COP profiles can be truncated for compositions toward the nonfeasible component.

The diversity of COP profiles demonstrates that the refrigerant set employed in our study encompasses a wide range of thermodynamic properties, allowing for general conclusions. Simultaneously, the COP profiles illustrate that finding optimal refrigerant mixtures is challenging and involves addressing multiple aspects from thermo-physical properties and equipment behavior (e.g., compressor) to process design.

### 3.2 Specialists among Refrigerant Mixtures

To map the efficiency advantage of zeotropic refrigerant mixtures, the highest refrigerant mixture COP is related to the highest pure refrigerant COP, COP_*ij*_^adv^ = COP_*ij*_^opt,mix^/COP_*ij*_^opt,pure^, for each pairing of heat source (*i*) and sink temperature changes (*j*) (Figure 5).

**Figure 5:**
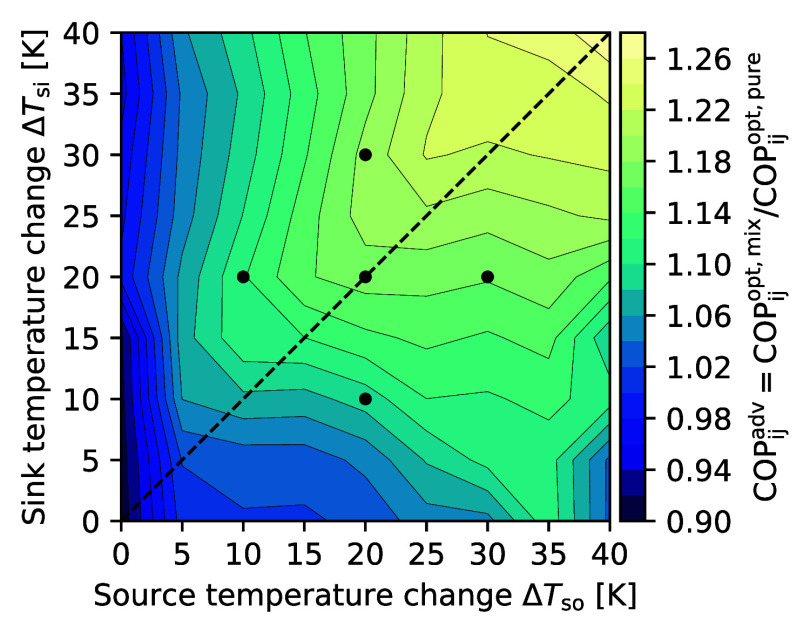
Mixture COP advantage COP^adv^ as a function of heat source and sink temperature change (*T*_so,in_ = 60 °C, *T*_si, out_ = 100 °C). The black dots refer to the heat source and sink pairings mentioned in the discussion (cf. Supporting Information for temperature-enthalpy diagrams, Figure SI3).

Refrigerant mixtures achieve higher COPs than pure refrigerants (COP^adv^ > 1), except for very low heat source temperature changes (Δ*T*_so_ ≤ 5 K). The advantage of mixtures COP^adv^ monotonically increases along the parity line (Δ*T*_so_ = Δ*T*_si_, black dashed line, Figure 5) toward larger temperature changes of heat source and sink. The refrigerant mixture COP advantage of 26% (COP_40,40_^adv^ = 1.26) is maximal for the heat source and sink pairing with the largest temperature changes (Δ*T*_so_ = Δ*T*_si_ = 40 K). The COP-optimal mixture for this heat source and sink pairing is cyclopentane (30 mol %)/cyclopropane (70 mol %) with COP = 4.4 corresponding to 69% of the reversible process COP_40,40_^rev^ = 6.3 (eq 2). The COP-optimal pure refrigerant cyclopropane, however, only achieves COP = 3.4.

The COP increase of refrigerant mixtures is attributed to the temperature glide: If the temperature glide matches the temperature changes of heat source and sink, the ratio of thermodynamic mean temperatures in the evaporator and condenser *T* _ev_/ *T* _co_ is increased, yielding higher COPs (cf. eq 2).^17^ If heat source temperature changes are small, mixtures with nearly no temperature glide are selected (quasi-azeotropic mixtures) and yield the highest COPs among all mixtures (Figure 6, left edge). However, they are still outperformed by pure refrigerants (Figure 5, left edge). The COP-optimal refrigerants along the parity line achieve glide matching with temperature glides similar to the temperature changes of both heat source and sink (Figure 6). Following basic thermodynamics, COP^adv^ increases toward large heat source and sink temperature changes (along the parity line, Figure 5) as the potential COP benefit of glide matching increases. Thus, the COP^adv^ is anticipated to further increase for even larger temperature changes of heat source and sink.

**Figure 6:**
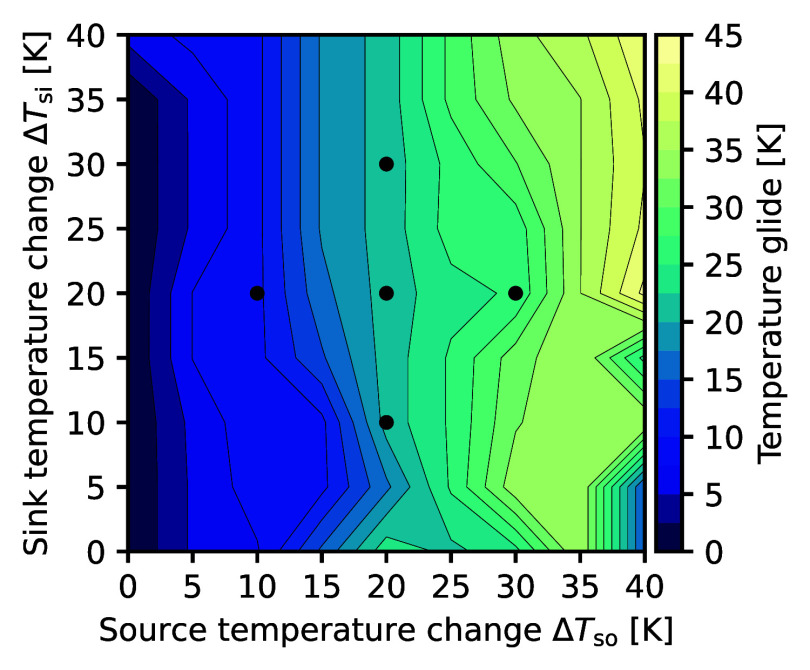
Temperature glide of the COP-optimal refrigerant mixture for every heat source and sink pairing. Temperature glide at a representative dew line temperature of *T*_dew_ = 60 °C. The black dots refer to the heat source and sink pairings mentioned in the discussion (cf. Supporting Information for temperature-enthalpy diagrams, Figure SI3).

However, the temperature changes of heat source and sink in industrial applications often differ, in particular heat source temperature changes are rather small for environmental heat sources (e.g., surface water) or low-temperature waste heat. Therefore, heat source and sink pairings off the parity line have particular significance for industrial applications. To analyze the influence of unequal temperature changes of heat source and sink, we start from a point of equal temperature changes (Δ*T*_so_ = Δ*T*_si_ = 20 K, middle dot in Figures 5 and 6) and move to unequal temperature changes in all directions (remaining dots in Figures 5 and 6). The associated heat pump processes are shown in temperature-enthalpy plots (Supporting Information, Figure SI3), which are arranged in the same configuration as the dots in Figures 5 and 6.

For Δ*T*_so_ = Δ*T*_si_ = 20 K the COP-optimal refrigerant mixture is 1-pentene (20 mol %)/cyclopropane (80 mol %), yielding a COP advantage of 17% (COP^adv^ = 1.17). The mixture’s temperature glide is 21 K (Figure 6), which closely matches the temperature changes of heat source and sink. For heat source and sink pairings with constant heat source temperature changes (vertically arranged dots, Figures 5 and 6), the COP remains nearly constant when the heat sink’s temperature change increases (upper dot) but decreases when the heat sink’s temperature change decreases (lower dot). Nevertheless, 1-pentene (20 mol %)/cyclopropane (80 mol %) remains the COP-optimal mixture; the mixture’s temperature glide of 21 K matches the heat source temperature change but is either too small or large for the heat sink temperature changes of Δ*T*_si_ = 30 K and Δ*T*_si_ = 10 K, respectively. Hence, glide matching in the evaporator (heat source) is more important for a high COP than glide matching in the condenser (heat sink). This trend is generally observed: The COP-optimal temperature glide follows the temperature change of the heat source (Figure 6).

The dominant influence of glide matching in the evaporator is attributed to a higher capability for cycle adjustments in the condenser (deheating, subcooling). Moreover, the two-phase heat transfer represents more of the entire heat transfer in the evaporator than in the condenser. In the evaporator, all heat is transferred during evaporation since superheating is performed in the IHX. Here, glide matching can significantly increase the thermodynamic mean temperature of evaporation and thus the COP.

In the condenser, only a part of the heat is transferred during condensation. Heat transfer during deheating and subcooling is not affected by glide matching (Figure 1), naturally limiting the temperature glide’s impact on decreasing the condenser’s thermodynamic mean temperature. The limited effect of glide matching in the condenser is further restricted by the narrow dome at close-to-critical temperatures. Furthermore, the subcooling increases flexibility to adapt the heat pump cycle to the heat sink. If the temperature glide is smaller than the heat sink’s temperature change, subcooling is increased to decrease the thermodynamic mean temperature and vice versa. However, the possibility of reducing the subcooling is limited (here: Δ*T*_sc,min_ = 3 K). If the temperature glide is still too large, the condensation temperature must be increased to ensure heat transfer. As a result, the COP decreases substantially.

For the heat source and sink pairings with constant heat sink temperature changes (horizontally arranged dots, Figure 5), the mixture COP advantage is almost constant when the heat source temperature change is increased (right dot) but reduced when the heat source temperature change is decreased (left dot). The COP-optimal temperature glide is aligned to the heat source’s temperature change (Figure 6) and achieved by distinct mixtures. Going from the middle dot (balanced heat temperature changes) to the left dot, the 1-pentene (20 mol %)/cyclopropane (80 mol %) is replaced by cyclobutene (60 mol %)/propylene (40 mol %). The COP-optimal mixture for the right dot is pentane (20 mol %)/cyclopropane (80 mol %).

An imbalance with a smaller temperature change in the heat source reduces the mixture COP advantage (left dot, Figure 5). The lower heat source temperature change generally decreases the glide matching COP potential in the evaporator. Still, the heat sink temperature change can be exploited by adequate subcooling, enhancing the mixture COP advantage achieved through glide matching.

For an imbalance with a larger temperature change in the heat source, the mixture COP advantage remains nearly constant (right dot, Figure 5). Even though the larger temperature change in the heat source benefits the mixture COP advantage, the resulting temperature glide is too large for the condenser, leading to an increase in the condensation temperature.

In summary, refrigerant mixtures yield significant COP advantages over pure refrigerants, with the benefit primarily driven by glide matching to the heat source temperature change. The mixture COP advantage is retained even with an imbalance between the temperature changes in the source and sink, provided that the heat source has a sufficiently large temperature change. An imbalance with higher temperature changes in the heat sink does not substantially affect the mixture COP advantage as glide matching in the condenser is less important and subcooling can be used to better match the temperature change of the heat sink. Extreme imbalances, however, can reduce the mixture COP advantage.

Discretizing the composition of mixtures by increments of d*x* = 0.1 mol/mol makes finding azeotropic points very unlikely and may contribute to the fact that no mixture COP advantage is found for low heat source temperature changes Δ*T*_so_ ≤ 5 K. Continuously optimizing the composition or choosing a higher resolution *dx* could potentially reveal COP advantages of azeotropic mixtures for quasi-isothermal sources due to mixture properties beyond the temperature glide. However, we estimate the advantage of azeotropic mixtures for increasing efficiency to be low and have excluded the analysis of azeotropic mixtures from our study.

COP-optimal specialist mixtures are highly diverse across all heat source and sink pairings: 21 refrigerant pairs are selected for the 81 heat source and sink pairings (full list in Supporting Information, Table SI3. Compare Figures SI1, SI2). Even though the COP is among the decisive criteria for refrigerant selection, such a highly diverse and specialized refrigerant portfolio is disadvantageous for manufacturers and operators of HTHPs: Highly specialized mixtures require custom-designed HTHPs, ensuring that all components are approved by the manufacturer for the particular mixture. In addition, regulations regarding safety and environmental protection must be observed, which are becoming increasingly complex.^34^ Consequently, when considering practical HTHP application in industry, refrigerants would be preferred that can be applied over a wide range of heat sources and sinks while still achieving near-optimal COPs.

### 3.3 All-Rounders among Refrigerant Mixtures

Moving on from the specialist refrigerant mixtures, we now search for versatile all-rounder refrigerants that yield high COPs over a wide range of industrial applications, represented by various heat source and sink temperature changes. With the composition as a degree of freedom, all-rounder mixtures promise high COPs while undercutting the effort associated with numerous application-specific refrigerants and can be a decisive step toward standardizing industrial high-temperature heat pumps.

The ranking of best all-rounder refrigerants across all heat source and sink pairings confirms the higher all-rounder potential of mixtures compared to pure refrigerants: The best all-rounder pure refrigerant ranks 73th (Table 5) and only achieves $\overset{®}{\mathrm{RCOP}}\approx 89\%$ (eq 5). The best all-rounder refrigerant pair is diethyl ether/cyclopropane with $\overset{®}{\mathrm{RCOP}}\approx 97\%$ . Beyond diethyl ether/cyclopropane, we find many more all-rounder refrigerant pairs enabling application across various heat source and sink pairings with only slightly decreased efficiencies compared to the respective COP-optimal refrigerants. However, refrigerant mixtures also can have significantly worse all-rounder performance than pure refrigerants (cf. lowest rank Table 5, isobutane/R1224yd­(Z)), exemplifying the need for careful selection.

**Table 5:** Ranking of $\overset{®}{\mathrm{RCOP}}$ (Eq 6) for All Refrigerants That Yield Feasible Heat Pump Cycles across All Heat Source and Sink Pairings Considered in the Screening Study^a^

		$\overset{®}{\mathrm{RCOP}}$	Δ*T*_glide_^max^	$\overset{®}{p_{\mathrm{low}}}$	$\overset{®}{p_{\mathrm{high}}}$	$\overset{®}{\mathrm{VHC}}$
rank	all-rounder refrigerant	[%]	[K]	[bar]	[bar]	[kJ/m^3^]
1.	diethyl ether/cyclopropane	97.0	35.7	3.2	16.2	3198.9
2.	1-pentene/cyclopropane	96.5	28.6	3.0	15.2	3030.6
3.	isopentane/cyclopropane	96.2	26.9	3.1	15.9	3093.9
4.	pentane/cyclopropane	96.1	34.0	2.6	12.5	2557.6
5.	1-pentene/dimethyl ether	95.6	30.2	3.0	15.8	3042.7
6.	cyclopentane/cyclopropane	95.5	47.8	3.0	14.3	2955.6
7.	cyclobutene/cyclopropane	95.2	9.3	3.3	20.6	3336.5
8.	diethyl ether/dimethyl ether	95.2	27.5	2.4	13.8	2597.0
9.	pentane/dimethyl ether	95.1	35.3	2.4	12.8	2531.8
10.	isopentane/dimethyl ether	95.0	28.4	2.5	14.2	2665.6
...						
73.	cyclopropane	89.2	0	5.5	32.9	4936.9
...						
152.	isobutane/R1224yd(Z)	83.2	9.7	2.2	16.4	2423.8

a The top ten refrigerants, the best pure refrigerant, and the worst refrigerant are displayed. Δ*T*_glide_^max^ represents the maximum temperature glide at a dew line reference temperature of 60 °C. $\overset{®}{p_{\mathrm{low}}}$ and $\overset{®}{p_{\mathrm{high}}}$ refer to the low and high pressure, $\overset{®}{\mathrm{VHC}}$ refers to the volumetric heating capacity of the refrigerant averaged across all case studies, respectively.

The best all-rounder pairs yield very similar near-optimal performance with close values for the Average Relative COP ( $\overset{®}{\mathrm{RCOP}}$ ). Similarities can be observed in the thermo-physical properties of the top-ranked all-rounder refrigerant pairs. The corresponding pure components typically exhibit properties that favor high COPs: high isentropic compressor efficiencies and evaporation enthalpies. Furthermore, the top-ranked all-rounder refrigerant pairs mostly have maximum temperature glides corresponding to the greater heat source and sink temperature changes in the case study (right column, Table 5). Tailoring the composition can then adapt the all-rounder’s temperature glide to most of the heat source and sink temperature changes. Simultaneously, the nonexcessive maximum temperature glides help avoid pinch point shifts in the heat exchangers and thus composition-dependent COP drops (Section 3.1).

The best-performing all-rounder refrigerant, diethyl ether/cyclopropane, yields near-optimal COPs for most heat source and sink pairings (Figure 7). Only for minimal source temperature changes (Δ*T*_so_ ≤ 5 K), the relative COP drops to around RCOP_5,5_ ≈ 91%. In this case, pure cyclopropane (*x*_mol_ = 1, Figure 9, left) is the most efficient in the diethyl ether/cyclopropane mixture but is outperformed by more efficient pure refrigerants (Table SI3). The COP-optimal compositions of diethyl ether/cyclopropane adapt the temperature glides to the respective heat source temperature changes (Figures 8 and 9, middle and right), exploiting the COP advantages achievable through glide matching.

**Figure 7:**
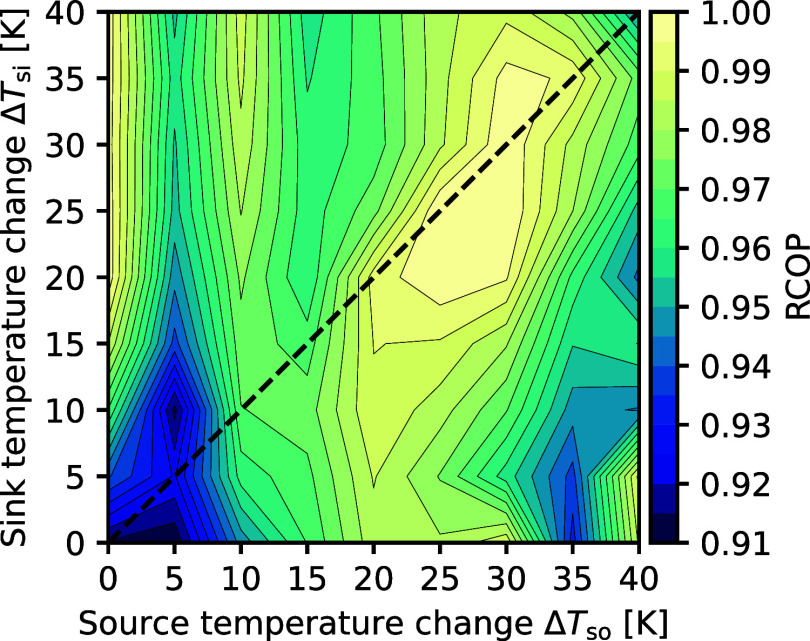
Relative COP (RCOP, eq 5) of diethyl ether/cyclopropane across all heat source and sink pairings.

**Figure 8:**
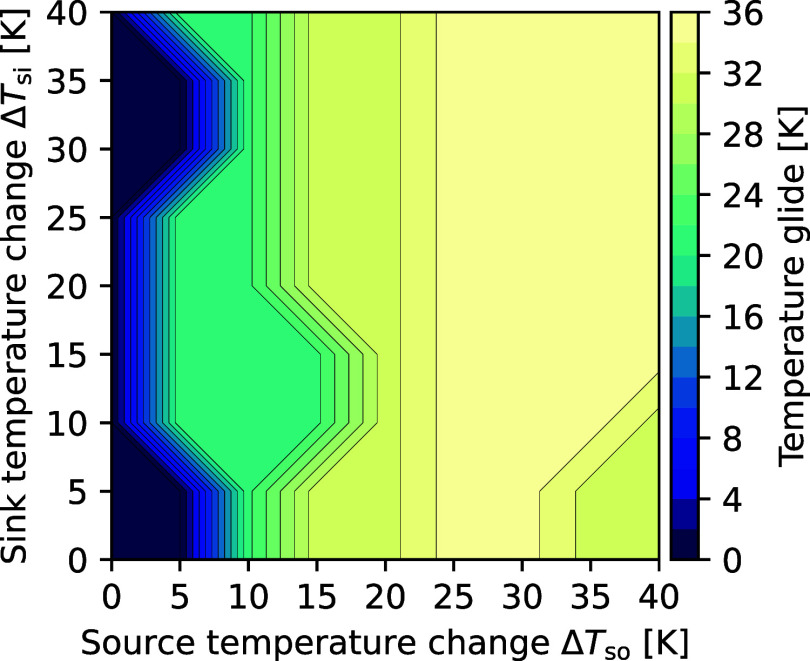
Temperature glide of diethyl ether/cyclopropane (at COP-optimal composition) at a dew line reference temperature of 60 °C.

**Figure 9:**
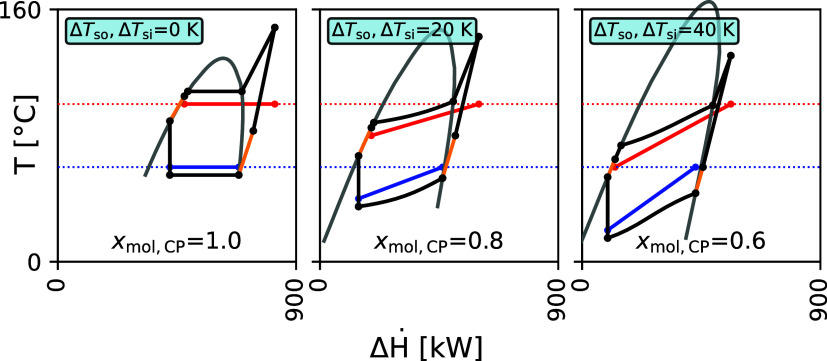
Temperature-enthalpy diagrams of the best-performing all-rounder refrigerant mixture, diethyl ether/cyclopropane, for three heat source and sink pairings (Δ*T*_so_, Δ*T*_si_) = (0 K, 0 K), (20 K, 20 K), (40 K, 40 K) (cf. parity line, Figure 7). Solid blue and red lines represent the heat source and sink, respectively. Heat sink outlet and heat source inlet temperatures are constant, indicated by the dashed red and blue lines. *x*_mol, CP_ indicates the molar fraction of cyclopropane.

From an application-oriented perspective, it may be interesting to find high-efficiency all-rounder refrigerants for a narrower range of heat source and sink temperature changes. In practice, applying the all-rounder concept to a specific subset of heat source and sink pairings (i.e., a manufacturer’s heat pump portfolio or an operator’s industrial heat pump applications) could increase process performance even further (shown in Supporting Information, Table SI4). Due to their higher all-rounder potential, refrigerant pairs outperform pure refrigerants for every subset of heat source and sink pairings.

All-rounder refrigerant mixtures are proposed to standardize HTHPs and, therefore, reduce total cost. By maintaining high COPs across various heat sources and sinks, all-rounder mixtures decrease the dominating operational costs in industrial applications (high annual operating hours). Additionally, all-rounder mixtures have the potential to reduce capital costs by minimizing the design effort, outclassing application-specific high-efficiency refrigerants (specialists): The all-rounder’s fixed pure components enable a basic heat pump design employing selected equipment (material compatibility, licensing). However, the presumed capital cost advantage of all-rounder mixtures depends on the cost and, therefore, the size of the equipment. To factor in equipment size/cost, the search for all-rounders must be expanded to selection criteria beyond cycle efficiency: Pressure levels and volumetric heating capacity (VHC) have a significant impact and vary considerably among all-rounder refrigerants (Table 5).

Because volumetric heating capacities vary, using a fixed compressor geometry results in different heating capacities depending on the refrigerant. A product design targeting a fixed heating capacity would require a refrigerant-specific compressor size selection. This study focuses on evaluating the all-rounder concept primarily with respect to efficiency. A fixed compressor geometry has limited impact on the analysis, as compressor efficiency is considered mainly dependent on the refrigerant properties rather than on compressor size.

The top all-rounder mixtures contain cyclopropane. The high COPs achievable with cyclopropane in HTHPs align with findings from current literature.^35,36^ However, chemical theory indicates low thermal stability for cycloalkanes due to the ring structure. Decomposition at high temperatures can produce noncondensable gases and deposits that reduce heat transfer rates, damage components, and compromise safety.^37^ Given the potentially high relevance of cycloalkanes as refrigerants in HTHPs, further research, particularly experimental results on thermal stability, can advance this field of study. Alongside mixtures featuring components that require further investigation, our screening identifies promising, ready-to-use all-rounder mixtures with components already tested in heat pumps such as 1-pentene/dimethyl ether.

## 4 Conclusions

This work aims to assess the potential of refrigerant mixtures for increasing efficiency and standardizing industrial high-temperature heat pumps (HTHPs). In the study, a representative set of 38 pure refrigerants was used to form 703 binary refrigerant pairs that were investigated across numerous compositions. A subcritical heat pump cycle with an internal heat exchanger was optimized for each refrigerant (pure + mixture) across 81 heat source and sink pairings, which were defined by systematic variations of temperature changes based on *T*_so,in_ = 60 °C and *T*_si,out_ = 100 °C.

We found the highest COPs for refrigerant mixtures with temperature glides closely matching the temperature changes of heat source and sink and high isentropic compressor efficiencies. Refrigerant mixtures are particularly advantageous compared to pure refrigerants for similarly large heat source and sink temperature changes. The maximum COP increase of a mixture compared to pure refrigerants of 26% was observed for the maximum temperature change in the heat source and sink (Δ*T*_so_ = Δ*T*_si_ = 40 K). Glide matching in the evaporator is the main driver for the increased COPs. Mixtures still yield a COP advantage over pure refrigerants in case there is an imbalance between the heat source’s and sink’s temperature changes, provided that the temperature change in the heat source is greater than 5 K.

We searched for all-rounder refrigerant pairs that yield near-optimal COPs across numerous heat source and sink pairings simply by adjusting the mixture composition. Diethyl ether/cyclopropane was the best all-rounder refrigerant pair, yielding on average 97% of the highest COP obtained by specialist mixtures. The all-rounder potential is clearly attributed to the additional degree of freedom provided by mixtures, namely the adjustment of the composition. Pure all-rounder refrigerants showed substantially lower COPs if used across various heat source’s and sink’s temperature changes.

In this study on the concept of all-rounder mixtures, simple equipment models such as pinch-based heat exchangers were used. For a dedicated techno-economic analysis, detailed physical equipment models should be applied that allow for sizing during optimization. Optimizing the heat pump process and equipment size toward a techno-economic objective (e.g., total cost) can provide insights into the economic feasibility of all-rounder mixtures. The sizing strategy can follow a worst-case approach, where the heat pump equipment is sized according to the most challenging heat source and sink for which the all-rounder is intended to be used. Alternatively, a modular approach can be chosen, where different sizes of basic equipment are selected depending on the all-rounder application. It is currently unclear how much differently sized equipment is required to cover the full working range, presenting an interesting optimization problem with economic trade-offs. These investigations must follow the fundamental introduction of the idea presented here.

Our study demonstrates the potential of refrigerant mixtures for high-temperature heat pumps. Refrigerant mixtures substantially increase the COP, particularly for applications with higher temperature changes in the heat source. Furthermore, refrigerant mixtures offer all-rounder potential, enabling the standardization of high-temperature heat pumps, which is a decisive step toward cost reduction and accelerated market introduction. We identified several promising binary mixtures that should be tested experimentally in future studies.

## Supplementary Material



## Notes

Additional details on the pure refrigerant set (Tables SI1, SI2, SI3), the COP-optimal specialist refrigerants (Figures SI1, SI2), and all-rounder refrigerants (pure + mixture) for various subsets of heat source and sink pairings (Table SI4). Selected aspects of the methodology (model equations, Section SI2; optimization, Section SI3) and discussion (glide matching, Section SI4) are compiled and presented concisely. The Supporting Information is intended to support the findings discussed in the main text by enhancing transparency and offering deeper context. (PDF)
