# Supplementary material for: One Mixture to Rule Them All: Enhancing Efficiency and Standardization of Industrial High-Temperature Heat Pumps
Source: ACS Eng Au. 2025 Jun 13;5(4):359–69. doi: 10.1021/acsengineeringau.4c00060 (PMC12371728; doi:10.1021/acsengineeringau.4c00060)
Supplement: Supplementary file 1 [file eg4c00060_si_001.pdf]

One Mixture to Rule Them All: Enhancing Efficiency and Standardization of Industrial High-Temperature Heat Pumps

Philip Widmaier[a], Leon P. M. Brendel[b], Stefan S. Bertsch[b], André Bardow[a], Dennis Roskosch[a,*]

–

Supplementary Information

[a]*Energy and Process Systems Engineering (EPSE), ETH Zurich, Tannenstrasse 3, 8092 Zurich, Switzerland*

[b]*Institute for Energy Systems (IES), Eastern Switzerland University of Applied Sciences, Werdenbergstrasse 4, 9471 Buchs, Switzerland*

*Corresponding author. Email: droskosch@ethz.ch

SI1. Main Text References

Table SI1: Isentropic compressor efficiencies η_{is} for all pure refrigerants considered in the screening study at $T_{\text{so,in}} = 60$ °C, $T_{\text{si,out}} = 100$ °C and $\Delta T_{\text{so}} = \Delta T_{\text{si}} = 0$ K. If the optimization routine can not determine a feasible cycle, the prevalent issue during randomized initial guess generation is listed (cf. optimization constrains, Table 2).

**Refrigerant**	**Isentropic compressor efficiency η_{is}**
1,3-butadiene	0.71
1-pentene	0.61
2,2-dimethylbutane	0.50
2,3-dimethylbutane	0.46
3-methylpentane	0.43
acetone	0.51
butane	0.70
butene	0.71
carbon dioxide	Subcritical process not possible
cyclobutene	0.71
cyclohexane	Compressor outlet temperature is too high
cyclopentane	0.53
cyclopropane	0.76
diethyl ether	0.60
dimethyl ether	0.75
ethane	Subcritical process not possible
ethanol	Compressor outlet temperature is too high
ethylene	Subcritical process not possible
hexane	0.39
isobutane	0.70
isobutene	0.71
isopentane	0.61
methane	Subcritical process not possible
methanol	Compressor outlet temperature is too high
pentane	0.58
propadiene	0.76
propane	Subcritical process not possible
propylene	Subcritical process not possible
R1224yd(Z)	0.59
R1233zd(E)	0.60
R1234yf	Subcritical process not possible
R1234ze(E)	Subcritical process not possible
R1234ze(Z)	0.63
R1243zf	Subcritical process not possible
R1336mzz(Z)	0.55
R152a	Minimum approach temperature can not be maintained
cis-butene	0.70
trans-2-butene	0.70

Table SI2: Pure refrigerant set used in the screening study.

**Refrigerant**	**Category**
ethanol	alcohols
methanol	alcohols
2,2-dimethylbutane	branched alkanes/alkenes
2,3-dimethylbutane	branched alkanes/alkenes
3-methylpentane	branched alkanes/alkenes
isobutane	branched alkanes/alkenes
isopentane	branched alkanes/alkenes
isobutene	branched alkanes/alkenes
cyclopropane	cycloalkane/-alkenes
cyclopentane	cycloalkane/-alkenes
cyclohexane	cycloalkane/-alkenes
cyclobutene	cycloalkane/-alkenes
diethyl ether	ethers
dimethyl ether	ethers
R152a	HFCs
R1224yd(Z)	HFOs
R1233zd(E)	HFOs
R1234yf	HFOs
R1234ze(E)	HFOs
R1234ze(Z)	HFOs
R1243zf	HFOs
R1336mzz(Z)	HFOs
butane	linear alkanes/alkenes
ethane	linear alkanes/alkenes
hexane	linear alkanes/alkenes
methane	linear alkanes/alkenes
pentane	linear alkanes/alkenes
propane	linear alkanes/alkenes
1-pentene	linear alkanes/alkenes
1-butene	linear alkanes/alkenes
cis-2-butene	linear alkanes/alkenes
ethylene	linear alkanes/alkenes
propylene	linear alkanes/alkenes
trans-2-butene	linear alkanes/alkenes
acetone	others
carbon dioxide	others
1,3-butadiene	other hydrocarbons
propadiene	other hydrocarbons

Table SI3: COP-optimal refrigerant mixtures and pure refrigerants for each heat source and sink pairing in the case study.

ΔT_{so}, ΔT_{si} [K]	**Refrigerant mixture**	**Pure refrigerant**
0, 0	cis-butene (10 mol-%)/cyclobutene (90 mol-%)	cyclobutene
0, 5	cis-butene (10 mol-%)/cyclobutene (90 mol-%)	cyclobutene
0, 10	cis-butene (10 mol-%)/cyclobutene (90 mol-%)	cyclobutene
0, 15	cis-butene (10 mol-%)/cyclobutene (90 mol-%)	cyclobutene
0, 20	cyclobutene (90 mol-%)/trans-butene (10 mol-%)	dimethyl ether
0, 25	cyclopropane (90 mol-%)/propadiene (10 mol-%)	dimethyl ether
0, 30	cyclopropane (90 mol-%)/propadiene (10 mol-%)	dimethyl ether
0, 35	dimethyl ether (10 mol-%)/propadiene (90 mol-%)	dimethyl ether
0, 40	dimethyl ether (10 mol-%)/propadiene (90 mol-%)	dimethyl ether
5, 0	cyclobutene (80 mol-%)/propadiene (20 mol-%)	cyclobutene
5, 5	cyclobutene (80 mol-%)/propadiene (20 mol-%)	cyclobutene
5, 10	cyclobutene (80 mol-%)/cyclopropane (20 mol-%)	cyclobutene
5, 15	cyclobutene (70 mol-%)/cyclopropane (30 mol-%)	cyclobutene
5, 20	cyclobutene (70 mol-%)/cyclopropane (30 mol-%)	dimethyl ether
5, 25	1,3-butadiene (30 mol-%)/cyclopropane (70 mol-%)	dimethyl ether
5, 30	1,3-butadiene (30 mol-%)/cyclopropane (70 mol-%)	dimethyl ether
5, 35	1,3-butadiene (30 mol-%)/cyclopropane (70 mol-%)	dimethyl ether
5, 40	propadiene (90 mol-%)/ethane (10 mol-%)	dimethyl ether
10, 0	acetone (20 mol-%)/cyclobutene (80 mol-%)	cyclobutene
10, 5	cyclobutene (60 mol-%)/cyclopropane (40 mol-%)	cyclobutene
10, 10	cyclobutene (50 mol-%)/cyclopropane (50 mol-%)	cyclobutene
10, 15	cyclobutene (50 mol-%)/cyclopropane (50 mol-%)	cyclobutene
10, 20	cyclobutene (50 mol-%)/cyclopropane (50 mol-%)	cyclopropane
10, 25	cyclobutene (50 mol-%)/cyclopropane (50 mol-%)	cyclopropane
10, 30	cyclobutene (50 mol-%)/cyclopropane (50 mol-%)	dimethyl ether
10, 35	cyclobutene (50 mol-%)/cyclopropane (50 mol-%)	dimethyl ether
10, 40	cyclobutene (50 mol-%)/cyclopropane (50 mol-%)	cyclopropane
15, 0	hexane (10 mol-%)/cyclobutene (90 mol-%)	cyclobutene
15, 5	cyclobutene (50 mol-%)/cyclopropane (50 mol-%)	cyclobutene
15, 10	cyclobutene (50 mol-%)/cyclopropane (50 mol-%)	cyclobutene
15, 15	pentane (10 mol-%)/cyclopropane (90 mol-%)	cyclobutene
15, 20	cyclobutene (60 mol-%)/propylene (40 mol-%)	cyclopropane
15, 25	cyclobutene (50 mol-%)/propylene (50 mol-%)	cyclopropane
15, 30	cyclobutene (50 mol-%)/propylene (50 mol-%)	cyclopropane
15, 35	cyclobutene (50 mol-%)/propylene (50 mol-%)	cyclopropane
15, 40	cyclobutene (50 mol-%)/propylene (50 mol-%)	cyclopropane
20, 0	pentane (20 mol-%)/cyclopropane (80 mol-%)	cyclobutene
20, 5	acetone (30 mol-%)/cyclobutene (70 mol-%)	cyclobutene
20, 10	1-pentene (20 mol-%)/cyclopropane (80 mol-%)	cyclobutene
20, 15	1-pentene (20 mol-%)/cyclopropane (80 mol-%)	cyclopropane
20, 20	1-pentene (20 mol-%)/cyclopropane (80 mol-%)	cyclopropane
20, 25	1-pentene (20 mol-%)/cyclopropane (80 mol-%)	cyclopropane

Table SI3 continued on the next page.

Table SI3 continued from the previous page.

ΔT_{so}, ΔT_{si} [K]	**Refrigerant mixture**	**Pure refrigerant**
20, 30	1-pentene (20 mol-%)/cyclopropane (80 mol-%)	cyclopropane
20, 35	1-pentene (20 mol-%)/cyclopropane (80 mol-%)	cyclopropane
20, 40	1-pentene (20 mol-%)/cyclopropane (80 mol-%)	cyclopropane
25, 0	cyclopentane (10 mol-%)/cyclopropane (90 mol-%)	cyclobutene
25, 5	acetone (10 mol-%)/cyclopropane (90 mol-%)	cyclobutene
25, 10	acetone (10 mol-%)/cyclopropane (90 mol-%)	cyclopropane
25, 15	acetone (10 mol-%)/cyclopropane (90 mol-%)	cyclopropane
25, 20	pentane (20 mol-%)/cyclopropane (80 mol-%)	cyclopropane
25, 25	diethyl ether (30 mol-%)/cyclopropane (70 mol-%)	cyclopropane
25, 30	1-pentene (30 mol-%)/cyclopropane (70 mol-%)	cyclopropane
25, 35	1-pentene (40 mol-%)/cyclopropane (60 mol-%)	cyclopropane
25, 40	1-pentene (40 mol-%)/cyclopropane (60 mol-%)	cyclopropane
30, 0	pentane (20 mol-%)/cyclopropane (80 mol-%)	cyclobutene
30, 5	cyclopentane (20 mol-%)/propadiene (80 mol-%)	cyclopropane
30, 10	acetone (20 mol-%)/dimethyl ether (80 mol-%)	cyclopropane
30, 15	2,3-dimethylbutane (10 mol-%)/cyclopropane (90 mol-%)	cyclopropane
30, 20	acetone (10 mol-%)/cyclopropane (90 mol-%)	cyclopropane
30, 25	diethyl ether (30 mol-%)/cyclopropane (70 mol-%)	cyclopropane
30, 30	pentane (30 mol-%)/cyclopropane (70 mol-%)	cyclopropane
30, 35	pentane (40 mol-%)/cyclopropane (60 mol-%)	cyclopropane
30, 40	pentane (40 mol-%)/dimethyl ether (60 mol-%)	cyclopropane
35, 0	cyclopentane (20 mol-%)/cyclopropane (80 mol-%)	cyclopropane
35, 5	cyclopentane (20 mol-%)/cyclopropane (80 mol-%)	cyclopropane
35, 10	cyclopentane (20 mol-%)/cyclopropane (80 mol-%)	cyclopropane
35, 15	cyclopentane (20 mol-%)/cyclopropane (80 mol-%)	cyclopropane
35, 20	cyclopentane (20 mol-%)/cyclopropane (80 mol-%)	cyclopropane
35, 25	cyclopentane (20 mol-%)/cyclopropane (80 mol-%)	cyclopropane
35, 30	cyclopentane (20 mol-%)/cyclopropane (80 mol-%)	cyclopropane
35, 35	cyclopentane (20 mol-%)/cyclopropane (80 mol-%)	cyclopropane
35, 40	isopentane (30 mol-%)/propylene (70 mol-%)	cyclopropane
40, 0	pentane (10 mol-%)/cyclopropane (90 mol-%)	cyclopropane
40, 5	pentane (10 mol-%)/cyclopropane (90 mol-%)	cyclopropane
40, 10	cyclopentane (20 mol-%)/cyclopropane (80 mol-%)	cyclopropane
40, 15	cyclopentane (30 mol-%)/dimethyl ether (70 mol-%)	cyclopropane
40, 20	acetone (20 mol-%)/propadiene (80 mol-%)	cyclopropane
40, 25	acetone (20 mol-%)/cyclopropane (80 mol-%)	cyclopropane
40, 30	acetone (20 mol-%)/propadiene (80 mol-%)	cyclopropane
40, 35	acetone (20 mol-%)/cyclopropane (80 mol-%)	cyclopropane
40, 40	cyclopentane (30 mol-%)/cyclopropane (70 mol-%)	cyclopropane

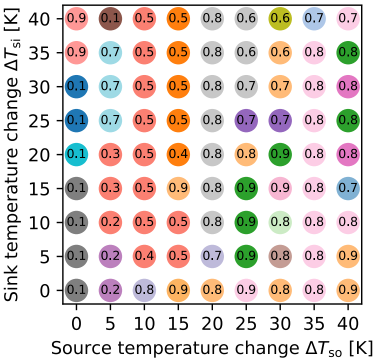

Figure SI1: Distribution of COP-optimal refrigerant mixtures across all heat source and sink pairings represented in the screening study. Each color represents a specific refrigerant pair, and annotations refer to mixture composition (second component's mole fraction, cf. Table SI3).

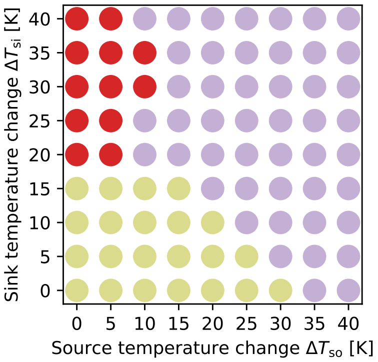

Figure SI2: Distribution of COP-optimal pure refrigerants across all heat source and sink pairings represented in the screening study. Each color represents a specific pure refrigerant (cf. Table SI3).

Table SI4: Average Relative COP ($\overline{\text{RCOP}}$, Equation 6) of the best all-rounder refrigerants for various subsets of heat source and sink pairings.
As subset all-rounder pure refrigerants, exclusively cycloalkanes (cyclobutene, cyclopropane) are identified.

Subset	Refrigerant pair		Pure refrigerant	
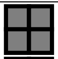	*Diethyl ether/Cyclopropane*	97.0%	*Cyclopropane*	89.2%
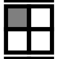	*Cyclobutene/Propylene*	98.6%	*Cyclopropane*	91.6%
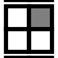	*Diethyl ether/Cyclopropane*	97.9%	*Cyclopropane*	83.0%
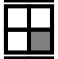	*Diethyl ether/Cyclopropane*	97.3%	*Cyclopropane*	89.6%
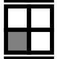	*Cyclobutene/Cyclopropane*	99.2%	*Cyclobutene*	94.6%
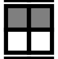	*Diethyl ether/Cyclopropane*	97.7%	*Cyclopropane*	87.6%
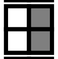	*Diethyl ether/Cyclopropane*	97.6%	*Cyclopropane*	86.4%
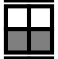	*Cyclobutene/Cyclopropane*	97.1%	*Cyclopropane*	90.8%
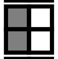	*Cyclobutene/Propylene*	98.7%	*Cyclobutene*	91.8%
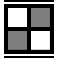	*Diethyl ether/Cyclopropane*	97.0%	*Cyclopropane*	87.4%
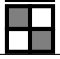	*Diethyl ether/Cyclopropane*	97.3%	*Cyclopropane*	90.7%

SI2. Model Equations

The process model's thermodynamic states (Table SI5) are calculated based on the evaporation and condensation temperatures ($T_{\mathrm{ev}}, T_{\mathrm{co}}$ defined at the dew line) and the superheating and subcooling ($\Delta T_{\mathrm{sh}}, \Delta T_{\mathrm{sc}}$). Process constraints are implemented to ensure a viable heat pump cycle (Table SI6).

Table SI5: Calculation of the process model's thermodynamic states based on the evaporation temperature T_{ev} and condensation temperature T_{co} (defined at the dew line, respectively), superheating ΔT_{sh}, and subcooling ΔT_{sc}.
This table corresponds to Table 1 (Section 2.1) from the main text.

**Thermodynamic State**	**State Parameters**	
1	$p_1 = p_{\mathrm{ev}} = p_{\mathrm{dew}}(T_{\mathrm{ev}})$	$T_1 = T_{\mathrm{ev}} + \Delta T_{\mathrm{sh}}$
2_{s}	$p_{2\mathrm{s}} = p_{\mathrm{co}} = p_{\mathrm{dew}}(T_{\mathrm{co}})$	$s_{2\mathrm{s}} = s_1$
2	$p_2 = p_{\mathrm{co}}$	$h_2 = \frac{h_{2\mathrm{s}} - h_1}{\eta_{\mathrm{is}}} + h_1$
3_{sc}	$p_{3\mathrm{sc}} = p_{\mathrm{co}}$	$T_{3\mathrm{sc}} = T_{\mathrm{bubble}}(p_{\mathrm{co}}) - \Delta T_{\mathrm{sc}}$
3	$p_3 = p_{\mathrm{co}}$	$h_3 = h_{3\mathrm{sc}} - h_1 + h_{\mathrm{dew}}(T_{\mathrm{ev}})$
4	$p_4 = p_{\mathrm{ev}}$	$h_4 = h_3$

Table SI6: Process constraints applied to the heat pump model.
R: refrigerant, SF: secondary fluid
This table corresponds to Table 2 (Section 2.3) from the main text.

**Constraint subject**	**Constraint**
Condensation temperature	$T_{\mathrm{co}} \leq T_{\mathrm{crit}} - 10$ K
Evaporator pressure	$p_{\mathrm{ev}} \geq p_{\mathrm{ev,min}} = 20$ kPa
Enthalpy during compression	$h_1 + \frac{h(p=p^*, s=s_1) - h_1}{\eta_{\mathrm{is}}} > h_{\mathrm{dew}}(p^*)$, $\forall p^* \in [p_{\mathrm{ev}}, p_{\mathrm{co}}]$
Approach temperature diff.	$\left\lvert T_{\mathrm{k}}^{\mathrm{R}} - T_{\mathrm{k}}^{\mathrm{SF}} \right\rvert \geq \Delta T_{\mathrm{app,min}} = 5$ K in all heat exchangers
Temperature lift	$T_{\mathrm{co}} > T_{\mathrm{ev}}$
Compressor outlet temperature	$T_2 < T_{2,\mathrm{max}} = 200$ °C

SI3. Optimization

The evaluation of refrigerants (pure + mixture) focuses on cycle efficiency and is based on the COP as the main performance metric (Equation SI1, main text Equation 1). The COP is optimized (Equation SI2, main text Equation 4) subject to process constraints (Table SI6) to determine the process parameters defining the most efficient heat pump cycle for each refrigerant (pure + mixture). Bounds are employed for each of the optimization parameters $T_{\mathrm{ev}}, T_{\mathrm{co}}, \Delta T_{\mathrm{sh}}, \Delta T_{\mathrm{sc}}$ to ensure sound optimization (Table SI7).

$$\mathrm{COP} = \frac{\dot{Q}_{\mathrm{h}}}{P_{\mathrm{comp}}} = \frac{q_{\mathrm{h}}}{w_{\mathrm{comp}}} \tag{SI1}$$

$$\begin{aligned} \max_{X} \quad & \mathrm{COP} = f(X) \\ \text{with} \quad & X = [T_{\mathrm{ev}}, T_{\mathrm{co}}, \Delta T_{\mathrm{sh}}, \Delta T_{\mathrm{sc}}] \\ \text{s.t.} \quad & h(X) = 0 \\ & g(X) \geq 0 \\ & X_{\mathrm{lb}} \leq X \leq X_{\mathrm{ub}} \end{aligned} \tag{SI2}$$

Table SI7: Optimization bounds for the process optimization variables.
This table corresponds to Table 3 (Section 2.3) from the main text.

	T_{ev}	T_{co}	ΔT_{sh}	ΔT_{sc}
Upper bound X_{ub}	$T_{\mathrm{so,in}}$	$T_{\mathrm{crit}} - 10$ K	28 K	28 K
Lower bound X_{lb}	$T(p = p_{\mathrm{ev,min}}, x = 1)$	$T_{\mathrm{si,in}}$	3 K	3 K

SI4. Mixture Efficiency Advantages through Glide Matching

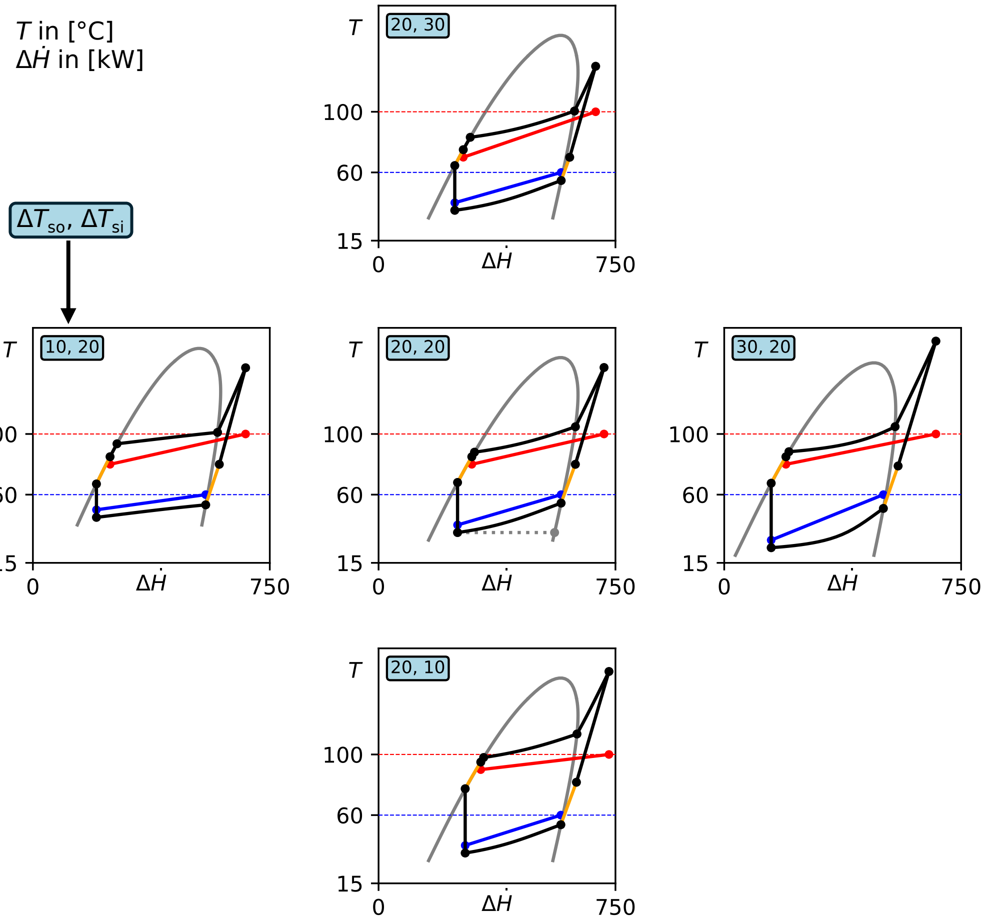

Figure SI3: Temperature-enthalpy diagrams of the COP-optimal refrigerant for five selected heat source and sink pairings $(\Delta T_{\mathrm{so}}, \Delta T_{\mathrm{si}}) = (20\text{ K}, 10\text{ K}), (20\text{ K}, 20\text{ K}), (20\text{ K}, 30\text{ K}), (10\text{ K}, 20\text{ K}), (30\text{ K}, 20\text{ K})$ (cf. black dots, Figures 5, 6). Solid blue and red lines represent the heat source and sink, respectively. Heat sink outlet and heat source inlet temperatures are constant, indicated by the dashed red and blue lines. The grey dotted line in the center diagram represents a pure refrigerant's evaporation.

The efficiency advantages of refrigerant mixtures over pure refrigerants are mainly attributed to glide matching (see Discussion, main text Section

3.2). Parts of the discussion are based on temperature-enthalpy diagrams specific to selected heat source and sink pairings (Figure SI.1) and thus are presented again for better comparability.

The temperature changes of heat source and sink in industrial applications often differ, in particular heat source temperature changes are rather small for environmental heat sources (e.g., surface water) or low-temperature waste heat. Therefore, heat source and sink pairings off the parity line have particular significance for industrial applications. To analyze the influence of unequal temperature changes of heat source and sink, we start from a point of equal temperature changes ($\Delta T_{\mathrm{so}} = \Delta T_{\mathrm{si}} = 20$ K, middle plot in Supplementary Information, Figure SI3) and move to unequal temperature changes in all directions (remaining plots in Figure SI3).

For $\Delta T_{\mathrm{so}} = \Delta T_{\mathrm{si}} = 20$ K the COP-optimal refrigerant mixture is 1-pentene (20 mol-%)/cyclopropane (80 mol-%), yielding a COP advantage of 17% ($\mathrm{COP}^{\mathrm{adv}} = 1.17$). The mixture's temperature glide is 21 K, which closely matches the temperature changes of heat source and sink. For heat source and sink pairings with constant heat source temperature changes (vertically arranged plots, Figure SI3), the COP remains nearly constant when the heat sink's temperature change increases (upper plot) but decreases when the heat sink's temperature change decreases (lower plot). Nevertheless, 1-pentene (20 mol-%)/cyclopropane (80 mol-%) remains the COP-optimal

mixture; the mixture's temperature glide matches the heat source temperature change but is either too small or large for the heat sink (compare vertically arranged plots, Figure SI3). Hence, glide matching in the evaporator (heat source) is more important for a high COP than glide matching in the condenser (heat sink). This trend is generally observed: The COP-optimal temperature glide follows the temperature change of the heat source.

The dominant influence of glide matching in the evaporator is attributed to a higher capability for cycle adjustments in the condenser (deheating, subcooling). Moreover, the two-phase heat transfer represents more of the entire heat transfer in the evaporator than in the condenser. In the evaporator, all heat is transferred during evaporation since superheating is performed in the IHX. Here, glide matching can significantly increase the thermodynamic mean temperature of evaporation (compare pure refrigerant evaporation (dashed line) and mixture evaporation (solid line), Figure SI3, middle plot) and thus the COP.

In the condenser, only a part of the heat is transferred during condensation. Heat transfer during deheating and subcooling is not affected by glide matching, naturally limiting the temperature glide's impact on decreasing the condenser's thermodynamic mean temperature. The limited effect of glide matching in the condenser is further restricted by the narrow dome at close-to-critical temperatures. Furthermore, the subcooling increases flexibil-

ity to adapt the heat pump cycle to the heat sink. If the temperature glide is smaller than the heat sink's temperature change, subcooling is increased (Figure SI3, top plot) to decrease the thermodynamic mean temperature and vice versa (Figure SI3, bottom plot). However, the possibility of reducing the subcooling is limited (here: $\Delta T_{\mathrm{sc,min}} = 3$ K). If the temperature glide is still too large, the condensation temperature must be increased to ensure heat transfer. As a result, the COP decreases substantially.

For the heat source and sink pairings with constant heat sink temperature changes (horizontally arranged plots, Figure SI3), the mixture COP advantage is almost constant when the heat source temperature change is increased (right plot) but reduced when the heat source temperature change is decreased (left plot). The COP-optimal temperature glide is aligned to the heat source's temperature change and achieved by distinct mixtures. Going from the middle plot (balanced heat temperature changes) to the left plot, the 1-pentene (20 mol-%)/cyclopropane (80 mol-%) is replaced by cyclobutene (60 mol-%)/propylene (40 mol-%). The COP-optimal mixture for the right plot is pentane (20 mol-%)/cyclopropane (80 mol-%).

An imbalance with a smaller temperature change in the heat source reduces the mixture COP advantage. The lower heat source temperature change generally decreases the glide matching COP potential in the evaporator. Still, the heat sink temperature change can be exploited by adequate

subcooling, enhancing the mixture COP advantage achieved through glide matching.

For an imbalance with a larger temperature change in the heat source, the mixture COP advantage remains nearly constant (right plot, Figure SI3). Even though the larger temperature change in the heat source benefits the mixture COP advantage, the resulting temperature glide is too large for the condenser, leading to an increase in the condensation temperature.

In summary, refrigerant mixtures yield significant COP advantages over pure refrigerants, with the benefit primarily driven by glide matching to the heat source temperature change. The mixture COP advantage is retained even with an imbalance between the temperature changes in the source and sink, provided that the heat source has a sufficiently large temperature change. An imbalance with higher temperature changes in the heat sink does not substantially affect the mixture COP advantage as glide matching in the condenser is less important and subcooling can be used to better match the temperature change of the heat sink. Extreme imbalances, however, can reduce the mixture COP advantage.
